# Estimates of Variance Components and Heritability Using Random Regression Models for Semen Traits in Boars

**DOI:** 10.3389/fgene.2022.805651

**Published:** 2022-02-04

**Authors:** Yifeng Hong, Limin Yan, Xiaoyan He, Dan Wu, Jian Ye, Gengyuan Cai, Dewu Liu, Zhenfang Wu, Cheng Tan

**Affiliations:** ^1^ College of Animal Science and National Engineering Research Center for Breeding Swine Industry, South China Agricultural University, Guangzhou, China; ^2^ National Engineering Research Center for Breeding Swine Industry, WENS Foodstuff Group Co., Ltd., Yunfu, China

**Keywords:** semen trait, random regression model, variance components, heritability, boars

## Abstract

It has been proven that the random regression model has a great advantage over the repeatability model in longitudinal data analysis. At present, the random regression model has been used as a standard analysis method in longitudinal data analysis. The aim of this study was to estimate the variance components and heritability of semen traits over the reproductive lifetime of boars. The study data, including 124,941 records from 3,366 boars, were collected from seven boar AI centers in South China between 2010 and 2019. To evaluate alternative models, we compared different polynomial orders of fixed, additive, and permanent environment effects in total 216 models using Bayesian Information Criterions. The result indicated that the best model always has higher-order polynomials of permanent environment effect and lower-order polynomials of fixed effect and additive effect regression. In Landrace boars, the heritabilities ranged from 0.18 to 0.28, 0.06 to 0.43, 0.03 to 0.14, and 0.05 to 0.24 for semen volume, sperm motility, sperm concentration, and abnormal sperm percentage, respectively. In Large White boars, the heritabilities ranged from 0.20 to 0.26, 0.07 to 0.15, 0.10 to 0.23, and 0.06 to 0.34 for semen volume, sperm motility, sperm concentration, and abnormal sperm percentage, respectively.

## Introduction

The use of artificial insemination (AI) is widely applied in the intensive pig industry as AI incredibly accelerates genetic progress by using semen of superior boars. However, young boars are seldom selected for AI considering semen traits. Even boars with high breeding values regarding production traits will still be culled due to poor semen quality. In addition to this, eliminative boars will receive a lower price in the commodity market (Lopez Rodriguez et al., 2017). Therefore, semen traits, such as semen volume, sperm motility, sperm concentration, and abnormal sperm percentage, affect the profitability of AI centers tremendously (Tesfay et al., 2020). So, adding semen traits to the selection index for boars at an early age not only can improve the profitability of AI centers but also increase female reproductive ability.

Some important economic traits of pigs, such as semen trait and body growth, are recoded with the age. In a few cases, the assumption of a repeatability model is invalid; however, a multivariate animal model would be highly overparameterized (Meyer and Hill, 1997). Random regression (RR) models were explained by Henderson (Henderson, 1982), and RR models are used to explain the repeated record and the longitudinal data that were collected multiple times for a single trait during the life time of animals (Hill, 1999).

In order to elaborate a reliable selection program for semen traits, estimating variance components and heritability for semen traits is important. Recently, more and more genetic parameters related to pig semen traits have been reported. However, both genetic and population diversity can influence the variance components. In addition, most of the studies related to semen traits are based on animal models or repeatability models (Wolf, 2009b; Marques et al., 2017; Li et al., 2019), and there is little research using random RR models for semen traits. Compared with animal models and repeatability models (Oh et al., 2006), the RR model demonstrated change of meaning and covariance along with age (Schaeffer, 2004). The RR model is not only widely used to estimate genetic parameters of milk yield in cows but also used for the analysis of growth data in pigs and beef cattle (Meyer, 1999; Andersen and Pedersen, 2010; Sasaki et al., 2017). The purpose of this study was to use an RR model to estimate genetic parameters of a series of semen traits, including semen volume, sperm motility, sperm concentration, and abnormal sperm percentage, in a large data set.

## Materials and Methods

Ethical review and approval were not required for the animal study because the data used for this study were collected as part of routine data that are recorded in a commercial breeding program. Semen collections were conducted strictly in line with the Guidelines for the Care and Use of Experimental Animals established by the Ministry of Science and Technology of the People’s Republic of China. All efforts were made to minimize animal suffering.

Data from seven AI centers of southern China were collected between 2010 and 2019. The total number of ejaculates was 124941 stemming from 3366 AI boars, including Landrace (LA, *n* = 1147) and Large White (LW, *n* = 2219). Semen volume (ml; VOL), sperm motility (%; MOT), sperm concentration (10 ^ 8/ml; CON), and abnormal sperm percentage (%; ABN) were considered in this study. VOL was measured by weighting each ejaculate and considering 1 g of semen to 1 ml. MOT, CON, and ABN were measured by a microscope (before 2017) and a computer-assisted sperm analysis system (after 2017).

Combined with previous studies and the characteristics of the data set, the following criteria are applied to data quality control: (a) the range age of boars between 33 and 150 weeks; (b) the first record was excluded, and the interval between two subsequent semen collections was within the range of 1–30 days; (c) animals with minimum ejaculation number (set to 6) were chosen to calculate the within-boar variation of the studies trait; (d) each fixed effect level should have at least 10 ejaculation records; (e) records on the VOL, MOT, CON, and ABN should be within 100 ml–600 ml, 10%–100%, 0.1 * 10^8/ml–8 * 10^8/ml, and 0.01–100%. After data filtering, the clean data of each breed are presented in Table1. These data and three-generation pedigree of boars were applied to the subsequent analysis.

**TABLE 1:** Number of boars and ejaculates for two breeds.

Breed^1^	Number of boars	Number of ejaculates	Ejaculates per boar	SD	Min^2^	Max^3^
LD	1147	38950	33.96	23.93	6	158
LW	2219	85991	38.75	26.38	6	150

Note: ^1^LD, Landrace; LW, Large White; ^2^Min = minimum number of records; ^3^Max = maximum number of records.

The following random regression model is used to estimate the (co) variance and breeding value:

$$y_{ijt}=AIYS_{i}+Interval_{j}+\sum_{l=0}^{nf}\varphi_{n}\left(w_{t}\right)\beta \_k+\sum_{l=0}^{nr}\varphi_{n}\left(w_{t}\right)a_{k}+\sum_{l=0}^{nr}\varphi_{n}\left(w_{t}\right)Pe_{k}+e_{ijt},$$

$y_{ijt}$ denotes the semen traits of boar recorded on day t within AIYS subclass i  and Interval subclass  j ; μ is the overall mean; $AIYS_{j}$ is the combined effects which include the AI center, year, and month; $Interval_{k}$ is the interval effect between two semen collections; $\beta_{k}$ is the fixed regression coefficients for the effect of the boar’s age; $a_{k}$ and $Pe_{k}$ are random regression coefficients for the additive genetic and permanent environmental effects, respectively, the terms $\varphi_{n}\left(w_{t}\right)$ correspond with Legendre polynomials evaluated at standardized time $w_{t}$ (-1 ≤ $w_{t}$ ≤ 1), and the residual is given by $\;e_{ijt}$ . The matrix of the model is accordingly denoted as follows: $y=X_{1}b_{1}+\;X_{2}b_{2}+\;Z_{1}a+\;Z_{2}p+e$ , where y is the vector of phenotypes; b_1_ is the vector of fixed effects; b_2_ is the vector of fixed regression coefficients; a and p are vectors of random regressions for additive genetic and permanent environmental effect, respectively, X_1_, X_2_, Z_1,_ and Z_2_ are design matrices of b_1_, b_2_, a, and p, respectively; and e is the vector of residuals. It was assumed that

$$\mathrm{var}\left[\begin{array}{c} a\\ p\\ e \end{array}\right]=\left[\begin{array}{ccc} A\sigma_{a}^{2} & 0 & 0\\ 0 & I\sigma_{p}^{2} & 0\\ 0 & 0 & I\sigma_{e}^{2} \end{array}\right],$$

where A is the numerator relationship, I is an identity matrix whose dimension is equal to the number of effect levels, $\sigma_{a}^{2}$ and $\;\sigma_{p}^{2}$ are co (variance) matrices of additive genetic and permanent environmental regression coefficients, respectively, and $\sigma_{e}^{2}$ stand for residual variance.

Legendre polynomials were generated using the following recursion formula:

$$\left(n+1\right)P_{n+1}\left(w_{t}\right)=\left(2n+1\right)w_{1}p_{n}\left(w_{t}\right)-nP_{n-1}\left(w_{t}\right),$$

where $P_{0}\left(w_{t}\right)$ = 1 and $P_{1}\left(w_{t}\right)=t$ . $P_{n}\left(w_{t}\right)$ is the polynomial of order n, and $w_{t}$ is the standardized time variable in the interval [-1,1] as $w_{t}=\;-1+\;\frac{2\left(a_{t}-a_{min}\;\right)}{a_{max}-a_{min}}$ , in which $a_{t}$ is the boar’s age when collecting semen traits, and $a_{min}$ and $a_{min}$ represent the first and latest boar’s age when collecting semen traits, respectively. The normalized value of the n^th^ Legendre polynomial evaluated at age t ( $\varphi_{n}\left(w_{t}\right)$ ) is as follows:

$$\begin{array}{l} \varphi_{0}\left(w_{t}\right)=0.7071;\\ \varphi_{1}\left(w_{t}\right)=1.2247t;\\ \varphi_{2}\left(w_{t}\right)=2.3717\left(t^{2}\right)-0.7906;\\ \varphi_{3}\left(w_{t}\right)=4.6771\left(t^{3}\right)-2.8067t;\\ \varphi_{4}\left(w_{t}\right)=9.2808\left(t^{4}\right)-7.9550\left(t^{2}\right)+0.7955;\\ \varphi_{5}\left(w_{t}\right)=18.4685\left(t^{5}\right)-20.5206\left(t^{3}\right)+4.3973t;\\ \varphi_{6}\left(w_{t}\right)=36.8085\left(t^{6}\right)-50.1935\left(t^{4}\right)+16.7312\left(t^{2}\right)-0.7967;\\ \varphi_{7}\left(w_{t}\right)=73.4291\left(t^{7}\right)-118.6162\left(t^{5}\right)+53.9164\left(t^{3}\right)-5.9907t;\\ \varphi_{8}\left(w_{t}\right)=146.5710\left(t^{8}\right)-273.5992\left(t^{6}\right)+157.8457\left(t^{4}\right)-28.992\left(t^{2}\right)+0.7972; \end{array}$$

We also used general linear models (GLMs) to perform the Waller−Duncan k-ratio *t*-test on the effects of semen collection interval. Models named L (a, b, and c) indicate the order of the polynomial fitted for fixed effects (a), additive genetic (b), and permanent environmental effects. This resulted in the evaluation of 200 models. Here, random regression models are fitted to be evaluated first through eighth-order polynomial covariance functions for the fixed effects of boar age classification and second through sixth-order polynomial covariance functions for the additive genetic and permanent environmental effects. The fitness of model was tested by Bayesian Information Criterions (BICs) (Neath and Cavanaugh, 2011):

BIC=−2log(L)+p×log(n);

where log(L) is the log-likelihood value, P stands for the number of parameters, and n is the sample size.

The semen trait change over time for all selected boars can be represented by $V=\;\varphi_{n}\left(w_{t}\right)\beta_{k},$ where V is a vector of actual semen traits from the boar’s age: 36 to 136 weeks, and others are same as before.

The estimate effects of the interval between two subsequent semen collections were also obtained from the abovementioned model.

## Results

**FIGURE 1:**
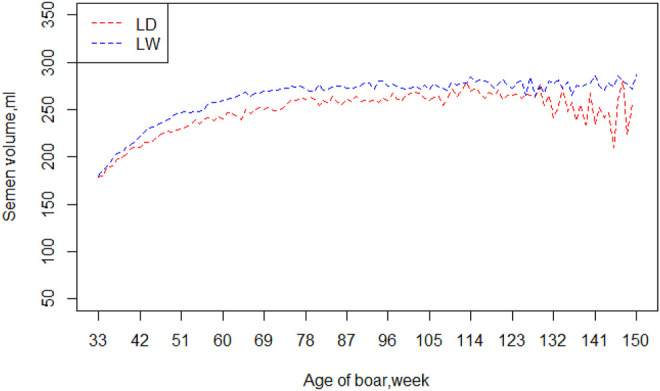
Mean of semen volume by age of boars in two breeds. Note: LD = Landrace, LW = Large White.

Table 2 exhibited a series of information, including means, standard deviations, and minimum and maximum of the four semen traits (VOL, DEN, MOT and ABN), for the two breeds. The means and standard deviation of semen volume were 240.62 and 77.44 ml in Landrace boars and 255.7 and 85.98 ml in Large White boars, respectively. The Large White boars had a larger mean of semen volume than Landrace boars. For the other three traits (DEN, MOT, and ABN), the Landrace boars and Large White boars had similar mean value and standard deviation. Figure 1 shows the average value of VOL in different ages of the two breeds. Large White boars always have a higher VOL value than Landrace boars over time, but their developing trends are similar. The 80th week is a turning point. Before 80 weeks, the means of semen volume increased followed with age, and it became stable after 80 weeks.

**TABLE 2:** Description statistics for semen traits in two breeds.

Breed^1^	Trait^2^	Mean	SD	Min3	Max4
LD	VOL	240.62	77.44	100	586
	MOT	68.68	12.01	10	100
	CON	3.23	1.96	0.1	8
	ABN	12.07	11.20	0.01	100
LW	VOL	255.7	85.98	100	600
	MOT	68.76	11.57	10	100
	CON	3.12	1.29	0.1	8
	ABN	12.03	10.17	0.01	100

Note:^1^LD, Landrace; LW, Large White; ^3^Trait: VOL, semen volume (ml), MOT, sperm motility (%), CON, sperm concentration (10^8/ml), ABN, abnormal sperm percentage (%), ^2^Min = minimum number of records; ^3^Max = maximum number of records.

Table 3 showed the order of fit for fixed (LF), additive genetic (LA), and permanent environmental (LP) effects; number of parameters (P), -2 times log likelihood (-2log(L), and Bayesian Information Criterions (BICs). The random regression model that fits LF = 4, LA = 3, and LP = 8 and LF = 5, LA = 4, and LP = 8 coefficients for fixed, additive genetic, and permanent environmental effects showed the smallest BIC for VOL in Landrace and Large White boars, respectively. For the MOT, LF = 3, LA = 3, LP = 8 and LF = 3, LA = 5, LP = 8 coefficients for fixed, additive genetic, and permanent environmental effects showed the smallest BIC in Landrace and Large White boars, respectively. In addition to this, LF = 7, LA = 5, LP = 5 and LF = 8, LA = 7, LP = 4 coefficients for fixed, additive genetic, and permanent environmental effects showed the smallest BIC for CON in Landrace and Large White boars, respectively. For the ABN, LF = 3, LA = 3, LP = 8 and LF = 4, LA = 5, LP = 7 coefficients for fixed, additive genetic, and permanent environmental effects showed the smallest BIC in Landrace and Large White boars, respectively. In conclusion, the low order of LA and LF and the high order of LP (especially LP = 8) best fits the RR model for VOL, MOT, and ABN, but not CON.

**TABLE 3:** Order of fit for fixed (L_F_), additive genetic (L_A_), and permanent environmental (K_p_) effects; negative twice of log likelihood (−2logL); Bayesian Information Criterions (BIC); and ranks of BIC.

Breed and trait^1^	Model 2	P3	-2Log(L)4	BIC5	Rank6
LD/VOL	L (4,3,8)	13	351983	352121	1
	L (5,3,8)	14	351975	352123	2
	L (6,3,8)	15	351965	352124	3
LD/MOT	L (3,3,8)	12	201527	201653	1
	L (3,4,8)	13	201522	201660	2
	L (3,5,8)	14	201523	201671	3
LD/CON	L (4,8,4)	14	38829	38977	2
	L (7,5,5)	15	38592	38751	1
	L (8,7,4)	17	39525	39705	3
LD/ABN	L (3,3,8)	12	198427	198552	1
	L (3,5,8)	14	198410	198558	2
	L (4,3,8)	13	198422	198560	3
LW/VOL	L (5,3,8)	14	775951	776110	2
	L (5,4,8)	15	775939	776109	1
	L (6,3,8)	15	775940	776111	3
LW/MOT	L (3,4,8)	13	436804	436952	2
	L (3,5,8)	14	436788	436947	1
	L (4,3,8)	13	436809	436957	3
LW/CON	L (8,4,5)	15	74339	74509	3
	L (8,7,3)	16	73553	73735	2
	L (8,7,4)	17	73451	73644	1
LW/ABN	L (3,4,8)	13	421778	421925	2
	L (3,5,8)	14	421767	421927	3
	L (4,5,7)	14	413477	413636	1

Note:^1^LD, Landrace; LW, Large white; VOL, semen volume (ml), MOT, sperm motility (%), CON, sperm concentration (10^8/ml), ABN, abnormal sperm percentage (%). ^2^L(a, b, and c) = the order of the polynomial fitted for fixed effects (a), additive genetic (b), and permanent environmental effects. ^3^P = the number of parameters in random regression models. ^4^Log(L) = log-likelihood value. ^5^BIC, Bayesian Information Criterions. ^6^Rank = The ranking of the top three models.

**FIGURE 2:**
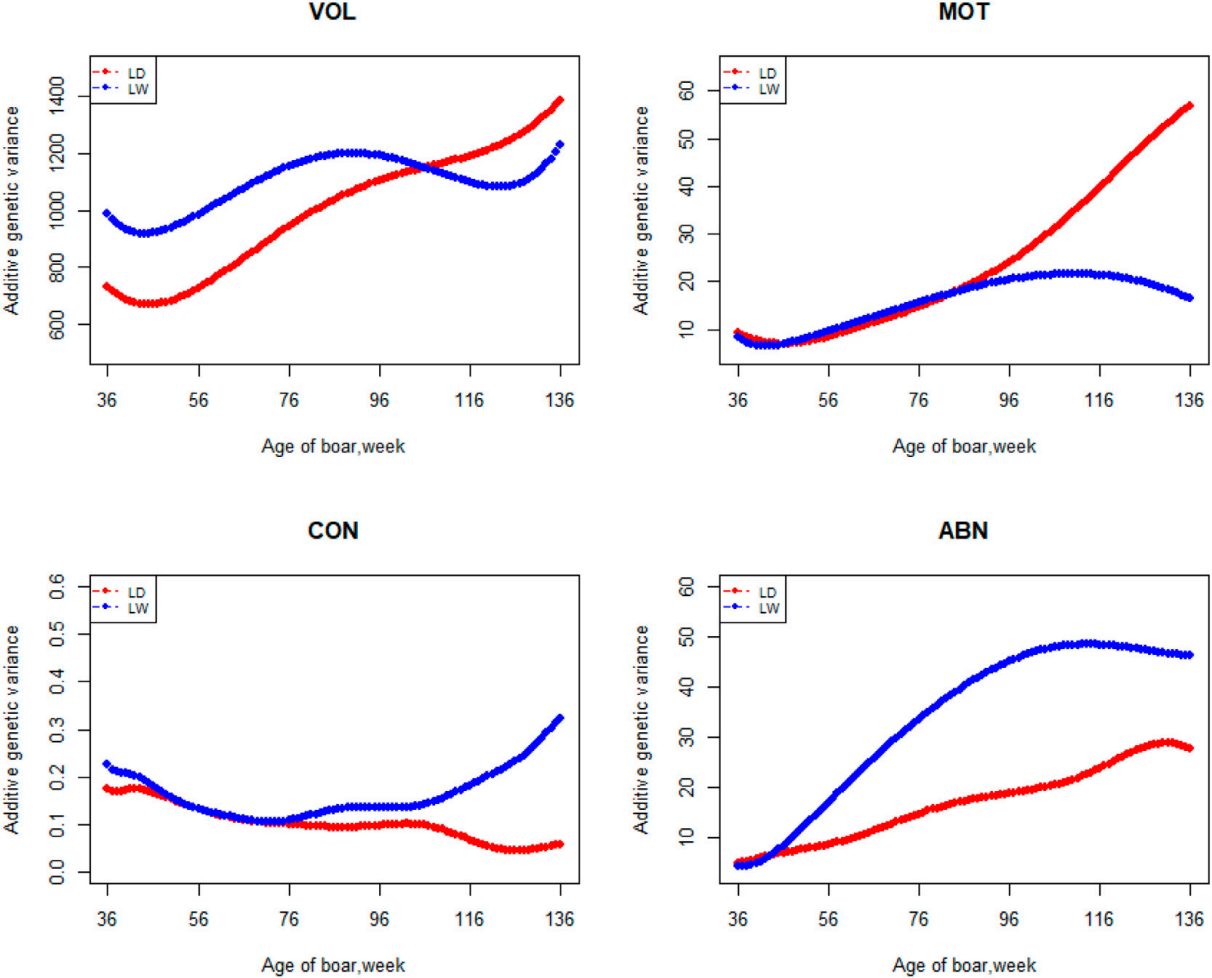
Additive genetic variance from 36 weeks to the age of 136 weeks, estimated with a random regression animal model.

**FIGURE 3:**
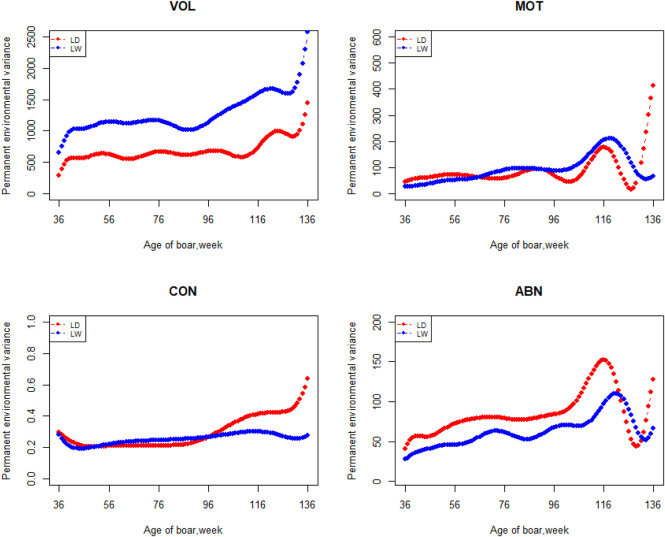
Permanent environmental variance from 36 weeks to the age of 136 weeks, estimated with a random regression animal model.

**FIGURE 4:**
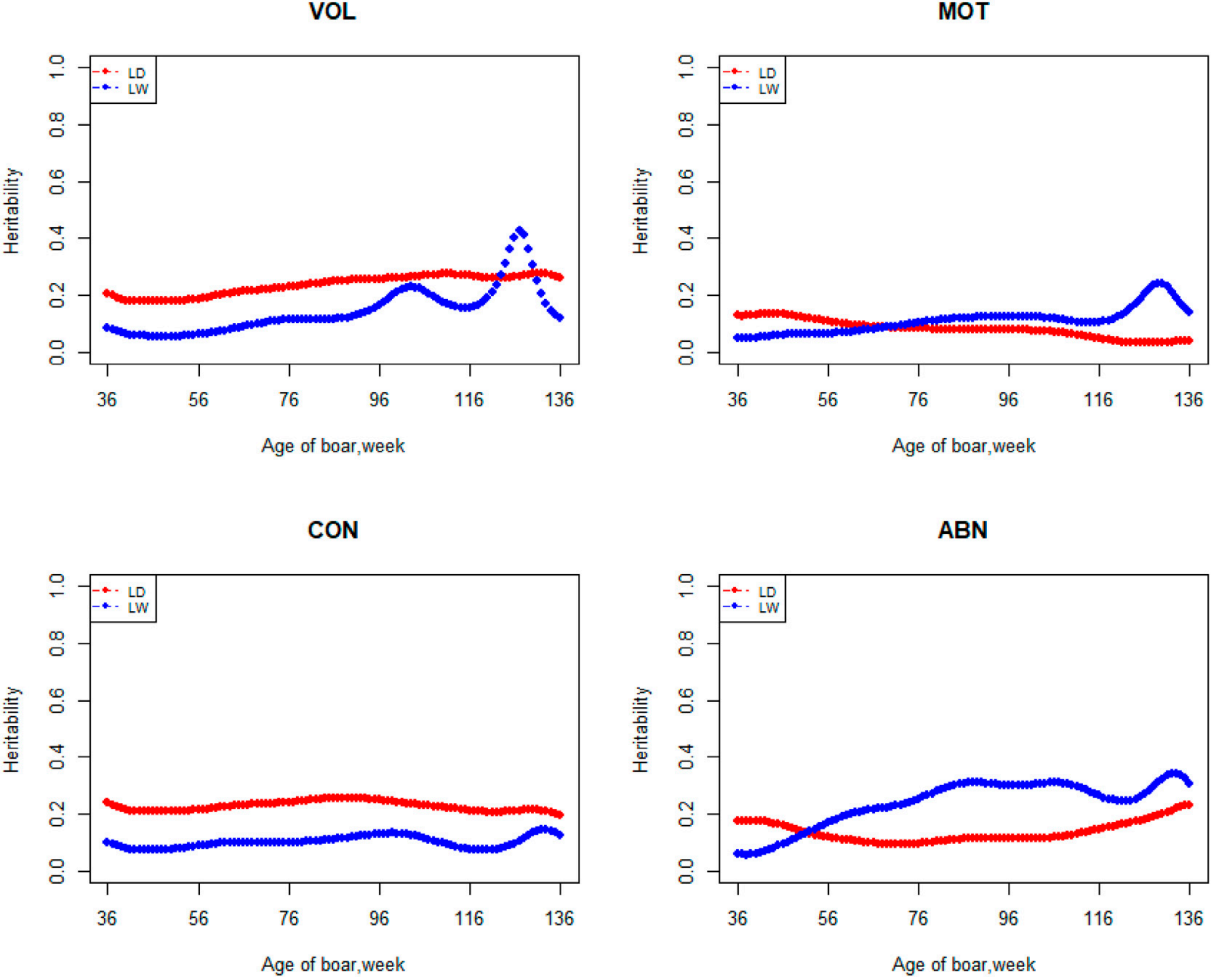
Heritability from 36 weeks to the age of 136 weeks, estimated with a random regression animal model.

**FIGURE 5:**
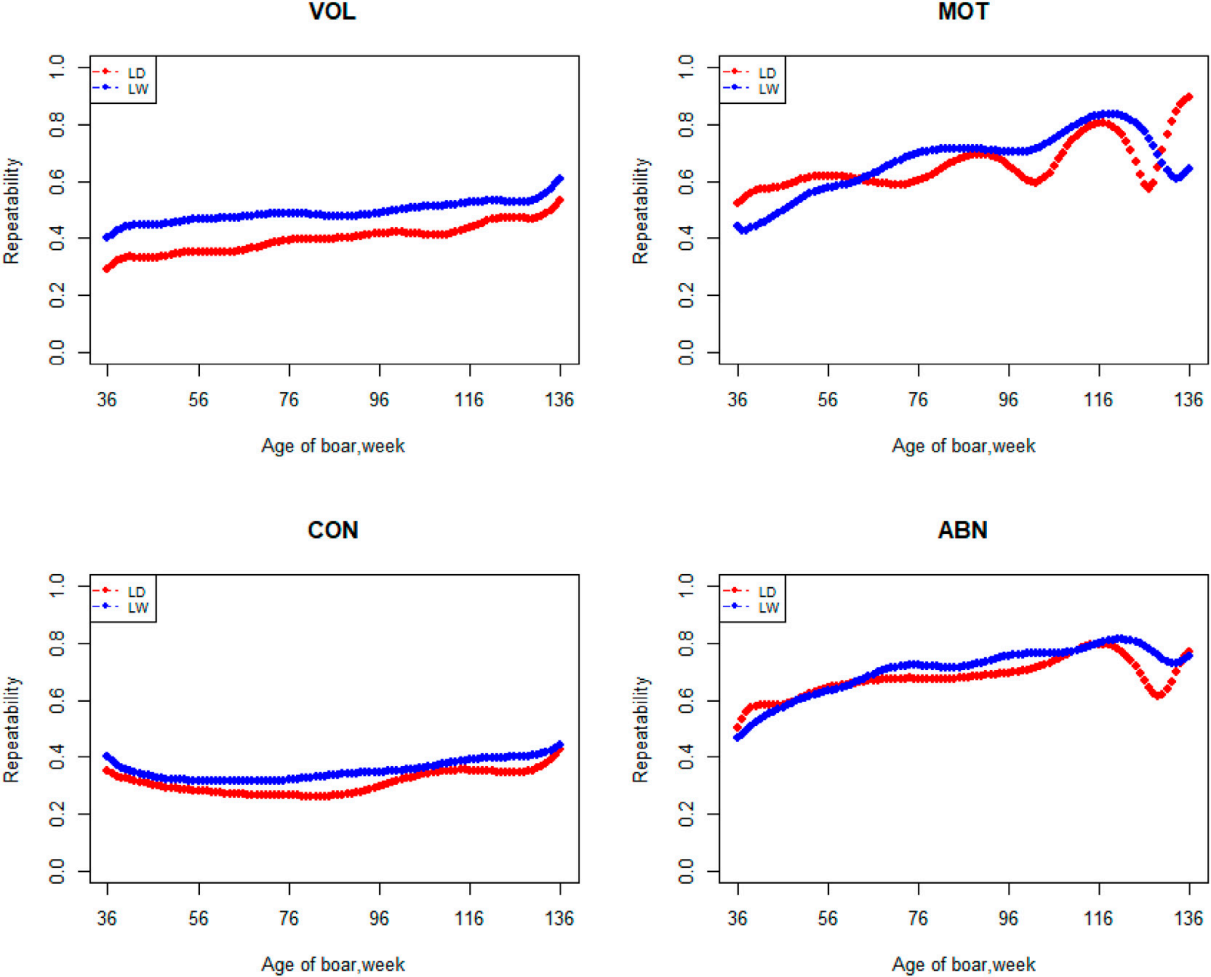
Repeatability from 36 weeks to the age of 136 weeks, estimated with a random regression animal model.

The variation of the additive genetic variance, permanent environmental variance, heritability, and repeatability for four semen traits in the two breeds changes over time as shown in Figures 2–5. In Landrace, the genetic variance, heritability, and repeatability estimates for VOL, MOT and ABN increased as the boar matured, while the CON decreased or remained stable along with time. In Large White, the additive variance and heritability estimates of ABN increase clearly as the boar matured, while the VOL constantly increase with age, and the additive variance and heritability for CON and MOT fluctuate somewhat over time. In summary, in Landrace, the additive variance ranged from 699.99 to 1384.12, 6.98 to 56.97, 0.05 to 0.17, and 4.81 to 28.73 for VOL, MOT, CON, and ABN, respectively. The permanent environmental variance ranged from 288.94 to 1440.16, 17.02 to 412.41, 0.20 to 0.64, and 41.16 to 152.06 for VOL, MOT, CON, and ABN, respectively. The heritabilities ranged from 0.18 to 0.28, 0.06 to 0.43, 0.03 to 0.14, and 0.05 to 0.24 for VOL, MOT, CON, and ABN, respectively. The repeatability ranged from 0.29 to 0.53, 0.52 to 0.89, 0.26 to 0.42, and 0.50 to 0.80 for VOL, MOT, CON, and ABN, respectively. In Large White, the additive variance ranged from 918.31 to 1228.71, 6.45 to 21.62, 0.11 to 0.32, and 4.15 to 48.41 for VOL, MOT, CON, and ABN, respectively. The permanent environmental variance ranged from 658.85 to 2568.19, 26.50 to 212.22, 0.19 to 0.30, and 28.08 to 110.30 for VOL, MOT, CON, and ABN, respectively. The heritabilities ranged from 0.20 to 0.26, 0.07 to 0.15, 0.10 to 0.23, and 0.06 to 0.34 for VOL, MOT, CON, and ABN, respectively. The repeatabilities ranged from 0.40 to 0.61, 0.43 to 0.83, 0.32 to 0.44, and 0.47 to 0.81 for VOL, MOT, CON, and ABN, respectively.

The genetic correlation estimate of VOL, MOT, CON, and ABN at different ages of boar is shown in Tables 4–7 respectively. Genetic correlations declined as the interval between ages increased in VOL and increased first and then increased in MOT, CON, and ABN. Genetic correlations of VOL, MOT, CON and ABN with the best random regression model from week 33 to week 150 range from 0.32 to 1, −0.52 to 1, −0.41 to 1, and 0.13 to 1 in Landrace. In Large White, the genetic correlation ranged from 0.06 to 1, 0.43 to 1, −0.33 to 1, and −0.13 to 1 for VOL, MOT, CON, and ABN in Landrace, respectively.

**TABLE 4:** Genetic correlations between semen volume of all ages, estimated with a random regression model.

Age week	33	42	51	60	69	78	87	96	105	114	123	132	141	150
33	−	0.96	0.85	0.73	0.6	0.5	0.42	0.35	0.29	0.24	0.19	0.14	0.1	0.06
42	0.96	−	0.97	0.89	0.8	0.72	0.65	0.59	0.52	0.46	0.38	0.29	0.19	0.09
51	0.85	0.96	−	0.98	0.93	0.87	0.82	0.77	0.71	0.64	0.55	0.43	0.28	0.13
60	0.72	0.89	0.98	−	0.99	0.96	0.92	0.88	0.83	0.76	0.66	0.53	0.36	0.18
69	0.6	0.8	0.93	0.99	−	0.99	0.97	0.94	0.9	0.84	0.74	0.61	0.43	0.23
78	0.51	0.73	0.88	0.96	0.99	−	0.99	0.98	0.94	0.89	0.8	0.67	0.49	0.28
87	0.44	0.67	0.84	0.93	0.98	1	−	0.99	0.97	0.93	0.86	0.73	0.56	0.35
96	0.4	0.63	0.8	0.9	0.96	0.98	1	−	0.99	0.96	0.9	0.79	0.63	0.43
105	0.36	0.59	0.76	0.87	0.93	0.96	0.98	0.99	−	0.99	0.95	0.86	0.71	0.53
114	0.35	0.56	0.72	0.83	0.89	0.93	0.95	0.98	0.99	−	0.98	0.92	0.81	0.64
123	0.34	0.53	0.67	0.77	0.83	0.87	0.91	0.94	0.97	0.99	−	0.98	0.9	0.77
132	0.33	0.49	0.61	0.69	0.74	0.79	0.83	0.87	0.91	0.95	0.99	−	0.97	0.89
141	0.33	0.44	0.53	0.59	0.63	0.67	0.72	0.77	0.82	0.88	0.94	0.98	−	0.97
150	0.32	0.39	0.43	0.47	0.5	0.54	0.58	0.64	0.71	0.78	0.86	0.93	0.98	

Note: Genetic correlations between semen volume of all ages between 33 and 150 weeks, estimated with a random regression model. Genetic correlations on the diagonal were Large White boars and under the diagonal were Landrace boars.

**TABLE 5:** Genetic correlations between sperm motility of all ages, estimated with a random regression model.

Age week	33	42	51	60	69	78	87	96	105	114	123	132	141	150
33	−	0.93	0.8	0.68	0.59	0.53	0.49	0.46	0.44	0.43	0.43	0.45	0.47	0.52
42	0.76	−	0.96	0.9	0.85	0.8	0.77	0.75	0.74	0.73	0.73	0.74	0.76	0.79
51	0.32	0.85	−	0.98	0.96	0.93	0.91	0.9	0.89	0.89	0.89	0.89	0.91	0.93
60	0	0.63	0.94	−	0.99	0.98	0.97	0.96	0.96	0.95	0.95	0.96	0.97	0.98
69	−0.22	0.43	0.82	0.97	−	1	0.99	0.99	0.98	0.98	0.98	0.98	0.99	1
78	−0.37	0.23	0.67	0.87	0.97	−	1	1	0.99	0.99	0.99	1	1	1
87	−0.47	0.05	0.49	0.74	0.88	0.97	−	1	1	1	1	1	1	1
96	−0.51	−0.1	0.32	0.59	0.77	0.9	0.98	−	1	1	1	1	1	1
105	−0.52	−0.2	0.18	0.45	0.65	0.81	0.93	0.99	−	1	1	1	1	1
114	−0.5	−0.26	0.09	0.35	0.57	0.74	0.88	0.96	0.99	−	1	1	1	0.99
123	−0.47	−0.27	0.05	0.31	0.52	0.71	0.85	0.94	0.98	1	−	1	1	1
132	−0.44	−0.24	0.07	0.31	0.52	0.7	0.85	0.94	0.98	1	1	−	1	1
141	−0.41	−0.18	0.14	0.38	0.58	0.75	0.88	0.95	0.99	1	1	1	−	1
150	−0.38	−0.07	0.27	0.5	0.68	0.82	0.92	0.97	0.99	0.98	0.98	0.98	0.99	−

Note: Genetic correlations between sperm motility of all ages between 33 and 150 weeks, estimated with a random regression model. Genetic correlations on the diagonal were Large White boars and under the diagonal were Landrace boars.

**TABLE 6:** Genetic correlations between sperm concentration of all ages, estimated with a random regression model.

Age week	33	42	51	60	69	78	87	96	105	114	123	132	141	150
33	−	0.95	0.86	0.74	0.58	0.44	0.32	0.21	0.07	−0.07	−0.19	−0.29	−0.33	−0.21
42	0.81	−	0.97	0.85	0.66	0.5	0.4	0.32	0.24	0.15	0.05	−0.06	−0.19	−0.25
51	0.62	0.96	−	0.95	0.8	0.66	0.58	0.52	0.47	0.4	0.3	0.2	0.06	−0.08
60	0.6	0.95	1	−	0.95	0.87	0.81	0.77	0.71	0.62	0.51	0.43	0.36	0.24
69	0.67	0.96	0.98	0.99	−	0.98	0.95	0.91	0.84	0.73	0.61	0.56	0.58	0.53
78	0.77	0.95	0.93	0.94	0.98	−	0.99	0.96	0.89	0.77	0.66	0.63	0.7	0.69
87	0.82	0.9	0.83	0.85	0.92	0.98	−	0.99	0.93	0.83	0.73	0.71	0.78	0.76
96	0.81	0.83	0.75	0.77	0.86	0.94	0.99	−	0.98	0.9	0.82	0.81	0.86	0.76
105	0.72	0.76	0.71	0.74	0.82	0.91	0.97	0.99	−	0.97	0.93	0.91	0.91	0.7
114	0.52	0.66	0.67	0.71	0.79	0.86	0.9	0.92	0.97	−	0.99	0.97	0.92	0.6
123	0.23	0.48	0.56	0.62	0.67	0.71	0.72	0.76	0.84	0.95	−	0.99	0.91	0.54
132	−0.01	0.23	0.34	0.4	0.45	0.49	0.53	0.58	0.69	0.85	0.96	−	0.94	0.59
141	0.02	−0.09	−0.09	−0.03	0.08	0.21	0.35	0.47	0.58	0.68	0.74	0.84	−	0.83
150	0.26	−0.24	−0.41	−0.39	−0.25	−0.04	0.16	0.29	0.33	0.27	0.18	0.26	0.73	−

Note: Genetic correlations between sperm concentration of all ages between 33 and 150 weeks, estimated with a random regression model. Genetic correlations on the diagonal were Large White boars and under the diagonal were Landrace boars.

**TABLE 7:** Genetic correlations between abnormal sperm percentage of all ages, estimated with a random regression model.

Age week	33	42	51	60	69	78	87	96	105	114	123	132	141	150
33	−	0.33	−0.04	−0.13	−0.13	−0.09	−0.05	−0.02	0.01	0.04	0.05	0.07	0.09	0.11
42	0.93	−	0.91	0.81	0.71	0.62	0.54	0.49	0.46	0.44	0.43	0.39	0.29	0.13
51	0.99	0.98	−	0.97	0.91	0.82	0.75	0.69	0.65	0.63	0.61	0.56	0.46	0.29
60	0.97	0.83	0.93	−	0.98	0.93	0.87	0.83	0.79	0.77	0.75	0.72	0.63	0.48
69	0.85	0.61	0.76	0.95	−	0.98	0.95	0.92	0.9	0.88	0.87	0.84	0.77	0.65
78	0.71	0.42	0.6	0.85	0.97	−	0.99	0.98	0.96	0.95	0.94	0.92	0.87	0.77
87	0.6	0.3	0.48	0.75	0.92	0.98	−	1	0.99	0.98	0.98	0.96	0.93	0.85
96	0.49	0.22	0.38	0.65	0.83	0.92	0.98	−	1	1	0.99	0.98	0.96	0.89
105	0.38	0.16	0.3	0.52	0.69	0.8	0.89	0.97	−	1	1	0.99	0.97	0.91
114	0.27	0.13	0.22	0.38	0.52	0.63	0.74	0.87	0.97	−	1	1	0.98	0.93
123	0.22	0.14	0.19	0.3	0.41	0.51	0.63	0.78	0.91	0.99	−	1	0.99	0.94
132	0.28	0.17	0.24	0.37	0.48	0.58	0.7	0.83	0.94	1	1	−	0.99	0.96
141	0.46	0.21	0.36	0.62	0.79	0.89	0.96	1	0.98	0.9	0.83	0.87	−	0.98
150	0.49	0.14	0.35	0.67	0.87	0.94	0.93	0.85	0.69	0.48	0.34	0.41	0.8	−

Note: Genetic correlations between abnormal sperm percentage of all ages between 33 and 150 weeks, estimated with a random regression model. Genetic correlations on the diagonal were Large White boars and under the diagonal were Landrace boars.

**FIGURE 6:**
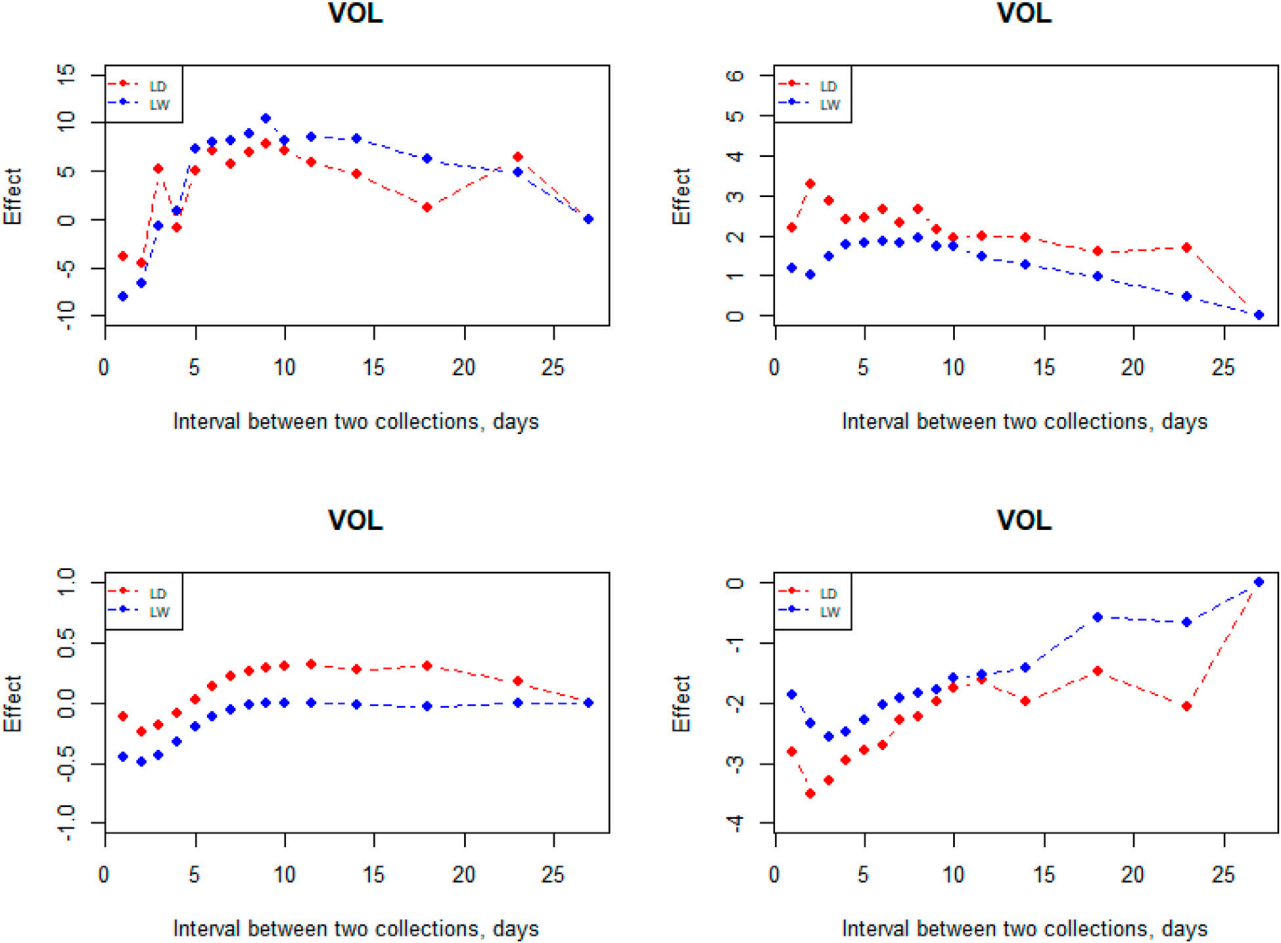
Interval between two subsequent semen collections on semen traits.

The interval between two subsequent semen collections on semen traits had a large effect (Figure 6). VOL increased when the interval was prolonged from 1 to 9 days; however, it decreases starting from the 10-day interval in the two breeds. In comparison, it is not an obvious effect in the interval between two subsequent semen collections on MOT, CON, and ABN. From the perspective of CON and ABN, the most suitable collective interval for Landrace is 2 days. When the semen collection interval is 9–10 days, the CON reaches the maximum in the two breeds.

## Discussion

It is very quick to obtain the result by using a repeatability model to analyze semen traits because of a substantially lower number of parameters (Gredler et al., 2007; Wolf, 2009a; Burren et al., 2019). However, the repeatability model assumes that two repeated measurements should have the same genetic correlations. Therefore, this model has some defects. First, the heritability of semen traits is different at different ages. Several researchers have reported that the heritability of daily milk yields is different from days in milk (Takma and Akbas, 2007). Second, genetic correlations among repeated measurements usually tend to decrease as functions of time. In comparison with the repeatability model, the RR models allow for modeling variance components as time functions, although the more parameters estimated, the more reasonable results. Using Legendre polynomials to fit RR models needs to be carried out carefully when interpreting results in extreme cases of boar age (Oh et al., 2006; Carabano et al., 2007). Li’s study shows that a random regression model with third-order of LP is suggested to be an appropriate model for genetic evaluation of milk yield in local Chinese Holstein populations (Li et al., 2020). However, the best model always has a higher order of permanent environment effect in the current study. Prakash discovered that the RR model with a lower-order polynomial for modeling additive genetic effect and higher-order polynomial for modeling animal permanent environmental effect is optimal for genetic evaluation (Prakash et al., 2017). In our study, a sudden increase in additive and permanent environmental variance relevant to the number of samples with over 136 weeks is small. Some studies reported that using splines to fit RR models was more robust against end of extreme time problems than polynomial models (Meyer, 2005; Bohmanova et al., 2008).

The best model indicated that VOL is a medium heritability trait with heritability ranging from 0.18 to 0.28 and from 0.20 to 0.26 in Landrace and Large White, respectively. These values strongly agreed with using the repeatability model for this trait reported previously by Wolf and Li (Wolf, 2009b; Li et al., 2019). Wolf estimated heritabilities of 0.19–0.25 for VOL in Czech Landrace, and Li estimated heritabilities of 0.25 ± 0.02, 0.21 ± 0.02, and 0.23 ± 0.02 for VOL in Duroc, Landrace, and Yorkshire, respectively. Heritability of VOL tends to increase and then decrease over time in Large White boars. Strathe reported a similar trend in heritability in the semen trait of pigs (Strathe et al., 2013). The heritability of sperm motility ranges from 0.06 to 0.43 and 0.07 to 0.15 in Landrace and Large White boars, respectively. Heritability changes suddenly after 96 weeks of age in the Landrace breed. It may due to insufficient data for Landrace boars, especially the data after 96 weeks. Marques reported that the heritabilities of MOT were 0.25 ± 0.05 and 0.08 ± 0.03 in Large White and Landrace boars, respectively. For the Large White, the heritability of MOT is higher than what we have studied, which may be due to the difference in population structure. Wolf estimated heritabilities of 0.06 ± 0.02 and 0.16 ± 0.03 for MOT in Czech Large White and Czech Landrace boars, respectively, and it is similar to our research. The heritabilities ranged from 0.03 to 0.14 and 0.10 to 0.23 for CON in Landrace and Large White boars, respectively. Grandjot estimated heritabilities of 0.17–0.26 (Grandjot et al., 1997) and Strathe estimated heritabilities of 0.23–0.26 for CON in Danish Landrace boars, which is slightly greater than the current estimates (Strathe et al., 2013). The heritabilities ranged from 0.05 to 0.24 and 0.06 to 0.34 for ABN in Landrace and Large White boars, respectively, which are well-consistent with those (0.15 ± 0.01 to 0.21 ± 0.02) obtained by Li and (0.15 ± 0.05 to 0.25 ± 0.06) estimated by Marques (Li et al., 2019; Marques et al.) using average value over all ejaculates for each boar and obtained estimates of 0.58, 0.38, 0.49, and 0.34 for VOL, MOT, CON, and ABN by Smital estimated, being substantially greater than the current estimates (Smital et al., 2005). This is reasonable because if the repeatability is less than 1, the heritability of the average number of records must be greater than that of a single record.

Apart from estimates of heritabilities, the estimates of the additive genetic variance and repeatability are also of particular interest to animal breeders. The additive variance directly determines the response to selection and the opportunities for genetic change by natural or artificial selection (Hill et al., 2008). For the VOL and ABN, the additive variance of Large White is obviously higher than that of Landrace in the first 100 weeks of age. The estimates of additive genetic variances for MOT and CON not differ greatly at the first 80 weeks of age. In the later stages of boar life, the additive variance usually varies a lot. It is determined by the character of models and the semen traits. In addition, fewer pigs survive as they age, leading to fewer records of high-frequency ejaculation. The repeatability of Large White is higher than that of Landrace in VOL and CON, but it showed fluctuation in the MOT and ABN.

As mentioned above, the estimate of heritability from four semen traits in Landrace and Large White indicated that selection for VOL could achieve reasonable rapid genetic gains. However, for the other three traits, the result indicated that the traditional selection will not gain genetic progress quickly because of low heritability. In addition, the boar semen traits are sex-limited traits, leading to the effect of traditional selection based on phenotype, and genealogical information is not obvious (Brigatti, 2021). Genome selection has outstanding advantages in complex traits and low heritability traits (Ibáñez-Escriche et al., 2014). How to estimate the breeding value of these traits and how to incorporate them into selectivity indicators will be considered in the next stage.

Genetic correlations between measurements at the age of 33 through 150 weeks are of great differences. Those results indicate that a repeatability model is an unacceptable approach to model variation for semen traits in this population. Genetic correlation decreases with age, which may also be due to limited data and selection of records in the prescribed age range. S.H.Oh estimated that genetic correlations were high between adjacent ages and decreased as the interval between ages increased in the sperm cell trait, and this result is consistent with our discovery (Oh et al., 2006). These results suggest that future performance may be harder to predict accurately from earlier records.

If the interval between ejaculation is too long, sperm function will be significantly reduced. However, if the interval between ejaculation is too short, the VOL will be significantly reduced (Check et al., 1991; Knecht et al., 2017). Thus, it is important to control the interval between successive collections. Based on the result of interval effect, it is indicated that 8–10 days is a best choice to design the interval during days of successive collection for Landrace and Large White breeds. However, it is not a best choice for MOT, CON, and ABN. Wolf found that the time interval of 7–10 days seems to be a good choice for getting the values of all semen traits near optimum (Wolf and Smital, 2009). Rutten et al. (2000) investigated collection intervals from 1 to 10 days and found that the highest number of doses per collection can be generated for intervals from 7 to 10 days. These results are in good agreement. Bajena reported that ejaculate CON remained at a relatively high level when ejaculates were collected with a frequency of 3–7 days, but further shortening of the interval between the successive collections led to a drastic decrease in CON (Bajena et al., 2016).

## Conclusion

We estimated the genetic parameters of VOL, MOT, CON, and ABN in different boar ages for two breeds. The higher-order polynomial of permanent environment effects and the lower-order polynomials for fixed effects and additive effects are the best orders to fit the random regression models. In addition, the best interval for semen collection is 8–10 days.

## Data Availability

The raw data supporting the conclusions of this article will be made available by the authors, without undue reservation.
